# Calcium binding protects E-cadherin from cleavage by *Helicobacter pylori* HtrA

**DOI:** 10.1186/s13099-016-0112-6

**Published:** 2016-06-06

**Authors:** Thomas P. Schmidt, Camilla Goetz, Markus Huemer, Gisbert Schneider, Silja Wessler

**Affiliations:** Cancer Cluster Salzburg, Department of Molecular Biology, Division of Microbiology, Paris-Lodron University, Salzburg, Austria; Department of Chemistry and Applied Biosciences, Swiss Federal Institute of Technology (ETH), Zurich, Switzerland

**Keywords:** *Helicobacter pylori*, HtrA, E-cadherin, Calcium, Protease

## Abstract

**Background:**

The cell adhesion and tumor suppressor protein E-cadherin is an important factor in the establishment and maintenance of epithelial integrity. E-cadherin is a single transmembrane protein, which consists of an intracellular domain (IC), a transmembrane domain (TD), and five extracellular domains (EC). EC domains form homophilic interactions in *cis* and *trans* that require calcium binding to the linker region between the EC domains. In our previous studies, we identified the serine protease high temperature requirement A (HtrA) from the human pathogen and class-I carcinogen *Helicobacter pylori* (*H. pylori*) as a bacterial E-cadherin-cleaving protease that targets the linker region of the EC domains, thereby disrupting gastric epithelial integrity. However, it remains unclear how calcium binding to the E-cadherin linker regions affects HtrA-mediated cleavage.

**Results:**

Investigating the influence of calcium on the HtrA-mediated cleavage of recombinant E-cadherin (rCdh1) in vitro, we tested different concentrations of calcium ions and the calcium chelator ethylenediaminetetraacetic acid (EDTA). Calcium efficiently reduced HtrA-mediated E-cadherin fragmentation. Conversely, the addition of EDTA strongly increased cleavage, resulting in a ladder of defined E-cadherin fragments. However, calcium ions did not affect HtrA oligomerization and protease activity as monitored by degradation of the universal protease substrate casein. Finally, addition of ethyleneglycol-bis-tetraacetic acid (EGTA) slightly enhanced E-cadherin cleavage during *H. pylori* infection of gastric epithelial cells.

**Conclusions:**

Our results suggest that calcium blocks HtrA-mediated cleavage by interfering with the accessibility of calcium-binding regions between the individual EC domains, which have been identified as cleavage sites of HtrA.

**Electronic supplementary material:**

The online version of this article (doi:10.1186/s13099-016-0112-6) contains supplementary material, which is available to authorized users.

## Background

**Fig. 1:**
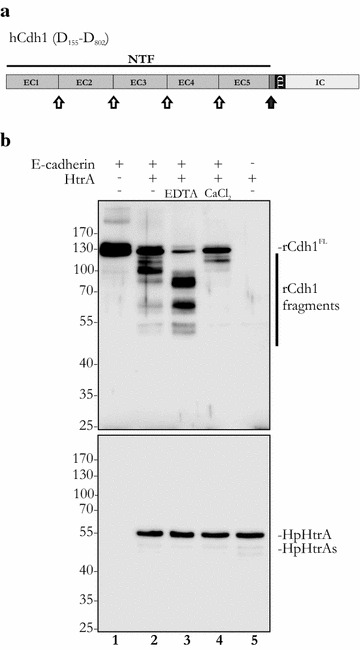
Calcium ions change the HtrA-mediated E-cadherin cleavage pattern. **a** Model of the domain structure of human E-cadherin (hCdh1). *EC* extracellular domain, *TD* transmembrane domain, *IC* intracellular domain, *NTF* soluble N-terminal fragment, *open arrows* signature sites for HtrA, *black arrow* cleavage site in the linker region. **b** 100 ng rCdh1 was incubated with 200 ng HpHtrA and, where indicated, with 125 µM EDTA or 100 µM CaCl_2_. Full-length E-cadherin (rCdh1^FL^, ~125 kDa) and rCdh1 fragments were detected by western blot using an antibody against the EC5 domain of E-cadherin. HpHtrA and the auto-processed short HpHtrA (HpHtrAs) were probed using a polyclonal antibody to show equal loading

The gastric mucosa provides important functions in the intake of nourishment and immunity, but also acts as an efficient barrier to mechanical and chemical influences as well as pathogenic microorganisms. The integrity of the protective epithelium is established by junctional complexes, including tight junctions, adherens junctions or desmosomes [1]. Epithelial cadherin (E-cadherin, Cdh1) is the key molecule of adherens junctions and is implicated in the establishment of intercellular adhesion and tumor suppression of human epithelia [2, 3]. Structurally, E-cadherin consists of an intracellular domain (IC), a single transmembrane domain (TD), and an extracellular domain (EC). The extracellular domain of E-cadherin is composed of five extracellular tandem repeats (called EC1–EC5) (Fig. 1a). Metal-binding motifs that bind calcium ions are located between the individual EC domains, and these are required for homophilic interactions between the domains. Upon calcium binding, E-cadherin changes its three-dimensional structure from a flexible conformation to a rigid, rod-like assembly favoring *trans*-interactions of EC domains 1 and 3. Further, calcium-binding has also been implicated in *cis*-oligomerization [4–11]. The intracellular domain of E-cadherin is bound by members of the catenin family, in particular β-catenin and catenin^p120^, which stabilizes E-cadherin-mediated intercellular adhesion. Through binding to E-cadherin, β-catenin bridges the IC domain to the actin cytoskeleton via direct binding to α-catenin or through interactions with additional factors, such as epithelial protein lost in neoplasm (EPLIN), myosin VI or vinculin [12–15]. Besides their important role in intercellular adhesion, intact E-cadherin-mediated adherens junctions function as significant tumor suppressors. Disruption of adherens junctions leads to a release of β-catenin and catenin^p120^ from the IC, which can then translocate into the nucleus where they interfere with the T cell factor/lymphoid enhancer factor (Tcf/Lef)-driven transactivation of cancer-associated genes (e.g. *c*-*myc*, *cyclin d1*) [16]. Importantly, there is a strong correlation between dysfunctional E-cadherin and cancer malignancy. Loss-of-function mutations, (epi) genetically downregulated *cdh1* expression or ectodomain cleavage influence the intercellular adhesion and the subcellular localization of associated catenins with severe consequences for cancer development and progression, particularly an increase in cellular invasiveness and metastasis [17].

E-cadherin also represents an attractive target for intruding human pathogens [18, 19]. Infections with the gastric pathogen and class-I carcinogen *Helicobacter pylori* (*H. pylori*) have been described to induce the disintegration of the E-cadherin complex in gastric epithelial cell lines leading to severe alterations in epithelial polarity [20–22]. *H. pylori* infections are widespread: an estimated 50 % of the world’s population is infected [23]. Although eradication of *H. pylori* with antibiotics is possible, its close association with the induction of duodenal and gastric ulcers, gastritis, mucosa-associated lymphoid tissue (MALT) lymphoma, and gastric cancer makes it a pathogen with a considerable impact on the health of its human host [24–26]. In recent studies, the bacterial serine protease high temperature requirement A (HtrA) was identified as a *H. pylori*-secreted factor that cleaves E-cadherin on gastric epithelial cells, thereby opening adherence junctions and disturbing the integrity of the gastric epithelial barrier [27]. HtrA proteases are chaperones and serine proteases that play important roles in protein quality control through their ability to refold and degrade misfolded proteins [28]. Members of the HtrA family of proteases have an N-terminal signal peptide, a protease domain and up to two PDZ (post synaptic density protein, *Drosophila* disc large tumor suppressor, zonula occludens-1 protein) domains. The PDZ domains regulate the protease function and oligomerization. For the *E. coli* homologue DegP, it has been shown that the hexamer presents the inactive state, whereas proteolytically active polyhedral cages (12, 24-mers) are formed for substrate degradation [28, 29]. However, DegP cage assembly and proteolytic activation can be uncoupled [30]. E-cadherin cleavage by prokaryotic HtrA proteases has also been described for several other gastrointestinal pathogens, such as enteropathogenic *Escherichia coli* (EPEC)*, Shigella flexneri* and *Campylobacter jejuni* [31–34], leading to the presumption that E-cadherin cleavage might be a general pathogenicity mechanism for gastric pathogens.

Although the cleavage events of E-cadherin on polarized gastric epithelial cell lines have been intensively investigated, detailed information about the molecular mechanisms of HtrA-mediated E-cadherin cleavage is still scarce. In our previous work, we identified the calcium-binding motifs as signature sites in the E-cadherin molecule that are preferentially targeted by *H. pylori* HtrA (HpHtrA) [35]. Interestingly, these signature sites are differentially accessible for HpHtrA, which might depend upon their homophilic interactions in *cis* and *trans* [35]. However, it remained completely unclear whether calcium binding affects HtrA activity and/or E-cadherin cleavage. Therefore, we have investigated the influence of calcium ions on the cleavage of E-cadherin by HpHtrA to provide a more detailed insight into the molecular mechanism through which HpHtrA interferes with E-cadherin functions.

## Results

### Depletion of calcium ions enhances HtrA-mediated E-cadherin cleavage

The bacterial protease HtrA secreted by *H. pylori* mediates efficient E-cadherin ectodomain (NTF, N-terminal fragment) shedding to open intercellular adhesion of polarized epithelial cells [27, 33]. We previously identified the residue motif [VITA]↓[VITA]-x-x-D-[DN] within the E-cadherin signature sites as preferred cleavage positions for HpHtrA [35]. These target sites are located between the individual EC domains (Fig. 1a, open arrows). Since these motifs are known to bind calcium ions [3, 5, 8], which are crucially important for the adhesive properties of E-cadherin, we investigated whether calcium ions affect HtrA-mediated cleavage of E-cadherin. According to the manufacturer´s instructions, recombinant E-cadherin (rCdh1) was reconstituted in phosphate-buffered saline (PBS), resulting in a final concentration of 45 µM CaCl_2_ in the in vitro cleavage reaction. Recombinant Cdh1 was incubated with HpHtrA for 16 h in vitro, and then E-cadherin fragmentation was analyzed by western blot using an antibody recognizing EC5. Full-length rCdh1 has a molecular weight of approximately 125 kDa (Fig. 1b, lane 1). Incubation with HpHtrA resulted in a partial cleavage. As previously described [35], a predominant ~100 kDa EC5-containing cleavage fragment and several minor products of ~90 kDa, ~60 kDa, and ~50 kDa were detected, while a large amount of full-length rCdh1 was still observed (Fig. 1b, lane 2). As a control, we also incubated rCdh1 with proteolytic inactive HtrA [36], which does not target E-cadherin (Fig. S1A, compare lanes 1 and 3). The addition of the EDTA and EGTA as calcium-chelating agents (Additional file 1: Figure S1A, lanes 9, 10) strongly increased E-cadherin processing by HtrA, leading to the formation of the ~90 kDa, ~60 kDa, and ~50 kDa fragments (Fig. 1b, lane 3). In contrast to this observation, an increase in the calcium ion concentration strongly reduced E-cadherin fragmentation. None of the 100 kDa, 90 kDa, 60 kDa, or the 50 kDa E-cadherin fragments was detectable anymore. Only a small amount of a 115 kDa truncated E-cadherin could be observed, indicating that calcium ions may block the accessibility of E-cadherin cleavage sites for HtrA (Fig. 1b, lane 4). Unlike CaCl_2_, MgCl_2_ did not influence E-cadherin cleavage (Additional file 1: Figure S1A, lanes 3–5). As further controls, we tested antibodies against E-cadherin (upper panel) or HtrA (lower panel) for possible cross-reactivity against HpHtrA (Fig. 1b, lane 5) or rCdh1 (Fig. 1b, lane 1), respectively. We detected a short HtrA (HpHtrAs) form (Fig. 1b), which has been previously identified by mass-spectrometry analyses as an auto-processed active form of HtrA [36].

### Calcium ions do not influence the oligomerization and proteolytic activity of HpHtrA

**Fig. 2:**
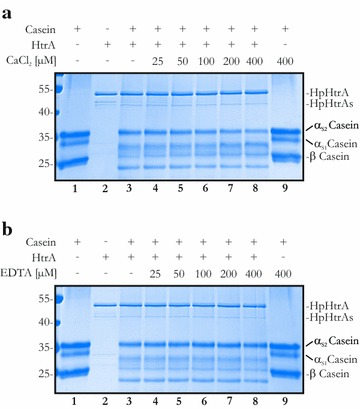
HtrA-mediated casein cleavage is independent of calcium ions. **a** 10 µg casein, composed of α_S1_-, α_S2_- and β-casein, was incubated with 200 ng HpHtrA and increasing concentrations of CaCl_2_ ranging from 25 to 400 µM as indicated. **b** Casein and HpHtrA were incubated with increasing concentrations of EDTA ranging from 25 to 400 µM. After 4 h incubation at 37 °C, samples were separated by SDS PAGE and proteins were stained with Coomassie G250. The original images of Coomassie-stained SDS gels are shown in Additional file 1: Figure S2

**Fig. 3:**
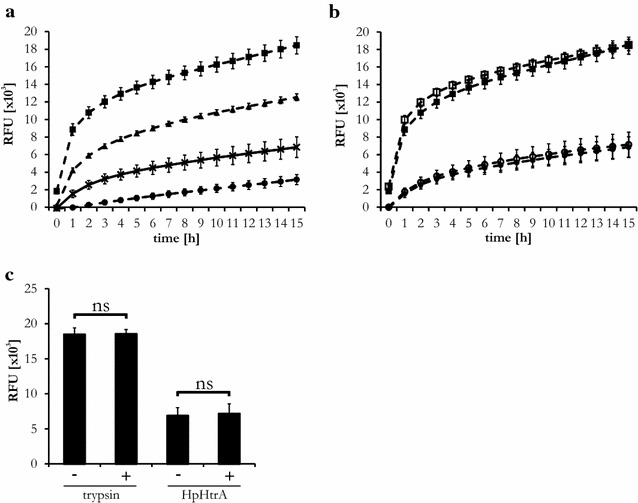
Calcium ions do not affect HpHtrA activity. **a** FITC-labeled casein was incubated with 1 µg/ml HpHtrA (*times*) and 0.008 µg/ml (*filled circle*), 0.2 µg/ml (*filled triangle*), and 1.0 µg/ml (*filled square*) trypsin as a positive control. **b** FITC-labeled casein was incubated with 1 µg/ml trypsin (*filled square*), 1 µg/ml trypsin/500 µM CaCl_2_ (*open square*), 1 µg/ml HpHtrA (*filled circle*), or 1 µg/ml HpHtrA/500 µM CaCl_2_ (*open circle*). Fluorescent signals of the blanks (casein ± CaCl_2_) were subtracted from the signals of casein with trypsin or HpHtrA. **c** Direct comparison of relative fluorescent signals of trypsin- or HpHtrA-treated FITC-labeled casein treated with 500 µM CaCl_2_ (+), as indicated, after 15 h. Data from three independent experiments are expressed as relative fluorescent units (RFU) ± SD

To analyze whether calcium affects HtrA activity, increasing concentrations of CaCl_2_ were incubated with HtrA and casein in vitro. Casein is composed of α_S1_-, α_S2_-casein and β-casein (Fig. 2a, lane 1) [37] and serves as an artificial substrate for monitoring the proteolytic activity of HtrA proteases [36]. After incubation of HpHtrA with casein for 4 h, the proteins were separated by SDS-PAGE and visualized by Coomassie staining (Fig. 2). The results indicate that HpHtrA preferentially cleaved α_S1_- and β-casein, which might be explained by the differences between these proteins in relation to hydrophobicity and post-translational modifications [37]. α_S2_-Casein was only targeted by HtrA at a low level (Fig. 2a, b, lane 3). Increasing concentrations of calcium ranging from 25 to 400 µM did not inhibit HtrA-mediated degradation of α_S1_- and β-casein. These observations were further confirmed by the observation that, after 16 h incubation, HpHtrA-mediated casein degradation was not inhibited by 1 mM CaCl_2_ or MgCl_2_ (Additional file 1: Figure S1B). In contrast, an obvious decrease in the level of α_S2_-casein was detectable in the presence of calcium, indicating a possible increase in HtrA activity and consequently induced casein degradation (Fig. 2a, lanes 4–8). These data were supported by the finding that addition of increasing amounts of EDTA did not alter HtrA-mediated α_S1_-, α_S2_- and β-casein cleavage (Fig. 2b). Since the results obtained from these in vitro cleavage assays showed no blocking or enhancement of HtrA activity by calcium after 4 h, we also monitored the kinetics of HtrA-mediated degradation of FITC-labeled casein to finally exclude an inhibitory effect of calcium ions on HtrA activity. As a positive control, increasing concentrations of trypsin were included, which led to a dose-dependent increase in the cleavage of the FITC-labeled casein (Fig. 3a). In comparison to 1 µg/ml trypsin, 1 µg/ml HpHtrA was less active, but induced an obvious casein degradation as reflected by the increase in relative fluorescent units (RFU) (Fig. 3a). Addition of 0.5 mM CaCl_2_ did not influence the proteolytic activity of HpHtrA during 15 h of HpHtrA-mediated casein degradation. A similar effect was detected for trypsin (Fig. 3a, c).

**Fig. 4:**
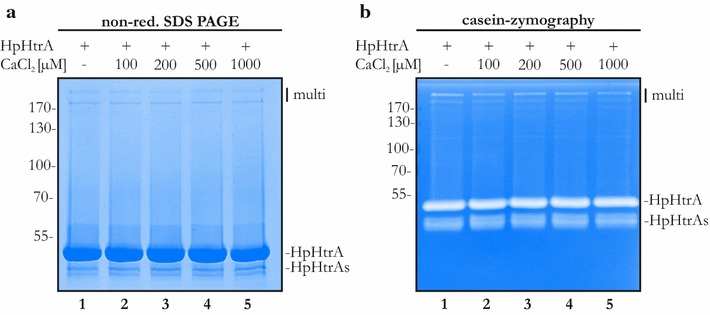
Calcium does not interfere with oligomerization of HpHtrA. **a** 3 µg HpHtrA were preincubated with increasing concentrations of calcium ions, as indicated, followed by separation by SDS-PAGE under non-reducing conditions. Proteins were Coomassie-stained to visualize HpHtrA in its monomeric and multimeric (multi) forms. **b** 200 ng HpHtrA were incubated with different concentrations of calcium ions for 16 h. Proteolytic activities of HpHtrA and HpHtrAs were analyzed by casein zymography

HpHtrA forms proteolytically active oligomeric structures [36]. *E. coli* DegP has been postulated to convert from an inactive to an active multimer upon substrate binding [28, 29]. Therefore, we aimed to test whether calcium can affect the oligomerization of HpHtrA. Separating HpHtrA by SDS-PAGE under non-reducing conditions clearly showed HpHtrA as a monomer and two additional multimeric structures (Fig. 4a, lane 1). This is in agreement with our previous studies showing that HpHtrA from worldwide strains can form oligomers [38]. Interestingly, the addition of increasing amounts of CaCl_2_ did not alter the formation of higher-structured oligomers (Fig. 4a, lanes 2–5). We made similar observations when analyzing the caseinolytic activity of HpHtrA in zymography experiments (Fig. 4b). The appearance of caseinolytically active HpHtrA migrating as a monomer and oligomer in the zymogram was independent of CaCl_2_ (Fig. 4b). These data support the hypothesis that calcium ions do not directly affect the proteolytic activity and oligomerization of HpHtrA.

### Calcium ions selectively inhibit HpHtrA-mediated E-cadherin cleavage

**Fig. 5:**
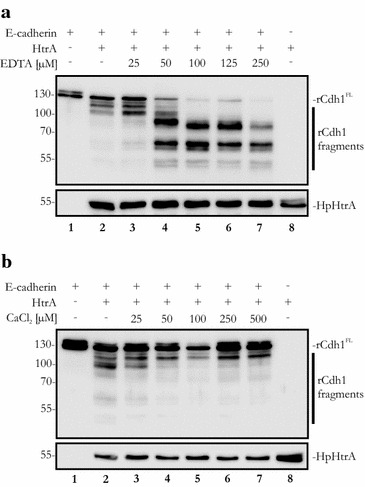
Influence of different CaCl_2_ and EDTA concentrations on the cleavage of recombinant E-cadherin by HpHtrA. 100 ng rCdh1 were incubated with 200 ng HpHtrA and, where indicated, with different concentrations of (**a**) EDTA or (**b**) CaCl_2_. Full-length rCdh1 (rCdh1^FL^) and rCdh1 fragments were detected by western blot using an antibody against the EC5 domain of E-cadherin. HpHtrA was probed with a polyclonal antibody to show equal loading of the protease

EDTA and calcium did not modify the activity of HtrA, but drastically interfered with E-cadherin cleavage. Therefore, we wanted to titrate the effects of EDTA and calcium on E-cadherin cleavage. Recombinant Cdh1 was incubated with HpHtrA together with increasing concentration of EDTA. At a concentration of 50 µM EDTA, a strong increase in E-cadherin fragmentation was observed (Fig. 5a, lane 4), which was further enhanced by increasing the EDTA concentration up to 250 µM (Fig. 5a, lanes 5–7). Correspondingly, only low concentrations of calcium ions were necessary to completely block HpHtrA-induced rCdh1 fragmentation. A concentration of 50 µM CaCl_2_ was sufficient to prevent the formation of the 100 kDa fragment (Fig. 5b, lane 4). A further increase in the CaCl_2_ concentration did not enhance the inhibition of E-cadherin cleavage, which was already limited to the generation of the 115 kDa E-cadherin fragment (Fig. 5b, lanes 5–7).

**Fig. 6:**
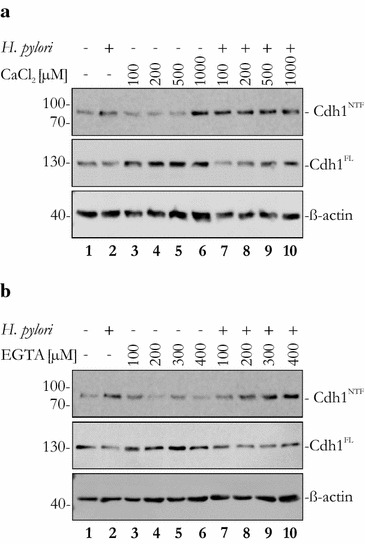
EGTA enhances E-cadherin shedding of *H. pylori*-infected NCI-N87 cells. NCI-N87 cells were infected with *H. pylori* at a MOI of 100 (+) for 16 h or left untreated (−). Cells were treated with increasing concentrations of (**a**) CaCl_2_ or (**b**) EGTA. E-cadherin fragments (Cdh1^NTF^) in the supernatant and full length E-cadherin (Cdh1^FL^) in whole cell lysates were detected by western blotting. β-actin was detected as a control

Calcium plays a crucially important role in the homophilic interactions of the extracellular domains of E-cadherin [5, 7–9]. We hypothesized that the accessibility of E-cadherin cleavage sites for HtrA might be regulated by the presence of calcium ions. To investigate the influence of calcium ions in *H. pylori*-mediated E-cadherin shedding, infected NCI-N87 cells were incubated with increasing concentrations of CaCl_2_ (Fig. 6a) or EGTA (Fig. 6b). Low concentrations of CaCl_2_ slightly decreased constitutive E-cadherin shedding through proteases [17], as reflected by the loss of NTF formation in the supernatants and the increase in full-length E-cadherin in whole cell lysates (Fig. 6a, lanes 3–5). However, 1 mM CaCl_2_ enhanced constitutive cleavage of E-cadherin, most likely through the activation of metalloproteases [39]. Increasing amounts of CaCl_2_ did not alter *H. pylori*-induced NTF formation (Fig. 6a, lanes 7–10) because the cell culture medium already contains 420 µM calcium to allow the formation of proper cell-to-cell adhesions via homophilic E-cadherin interactions. Subsequently, cells were treated with increasing concentrations of EGTA, which led to a slight increase in NTF in supernatants of EGTA-treated cells (Fig. 6b). Higher concentrations of EGTA to efficiently complex Ca^++^ in the medium result in dislocation and malfunction of E-cadherin [40] and were not included in this study. In conclusion, our data suggest that binding of calcium ions to E-cadherin masks cleavages sites for HpHtrA in vitro and on epithelial cells, thereby providing a tightly controlled mechanism of E-cadherin shedding.

## Discussion

**Fig. 7:**
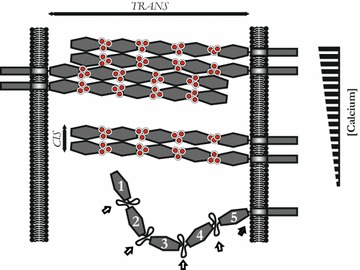
Model of calcium-regulated HpHtrA-mediated cleavage of E-cadherin. The extracellular N-terminal part of E-cadherin is shown with the identified signature sites (*open arrows*) between the five EC domains (EC1-EC5) and an additional cleavage site in the linker region (*black arrow*) for HpHtrA. Signature sites can be bound by calcium ions (*red spheres*) leading to conformational changes in the E-cadherin molecule, which allow homophilic interactions of E-cadherin in *cis* and *trans*. HtrA-mediated E-cadherin cleavage can be inhibited after binding of calcium ions to the signature sites, either by blocking the accessibility of signature sites for HpHtrA or by changing of the conformational structure of E-cadherin

The controlled cleavage of E-cadherin is a fundamental process in the pathogenesis of *H. pylori* [20, 27, 41] which could play a crucially important role in gastric carcinogenesis and metastasis. The bacterial serine protease HtrA has been identified as an E-cadherin-targeting protease that directly cleaves off the extracellular domain of E-cadherin to release the soluble 90 kDa NTF into the culture supernatant [27, 33]. Investigations of the different E-cadherin fragmentation patterns upon *H. pylori* infection and in in vitro cleavage experiments have revealed the existence of signature sites for HpHtrA in the E-cadherin molecule (Fig. 7, open arrows) and an additional cleavage site between EC5 and the transmembrane domain (Fig. 7, black arrow). These signature sites are directly targeted in vitro, leading to the formation of a defined fragmentation pattern, which is in contrast to the observed stable 90 kDa EC5-containing NTF detected after infection of gastric epithelial cells [35]. In our current model, the identified HtrA-targeted signature sites are not accessible on epithelial cells through the formation of functional homophilic interactions of the extracellular domain in *cis* and *trans*, which might explain the production of the stable 90 kDa NTF upon *H. pylori* infection [35].

Since the HtrA signature sites harbor the binding motifs for calcium, we aimed to investigate whether calcium ions affect the E-cadherin cleavage pattern and found that addition of Ca^2+^ selectively blocked HtrA-mediated E-cadherin cleavage, but did not interfere with the proteolytic activity of HpHtrA *per se*. The effect of certain divalent cations on proteases of the HtrA family of other species has recently been investigated. It was shown that the activity of *Borrelia burgdorferi* HtrA (BbHtrA) is inhibited by Zn^2+^, Mn^2+^, and Cu^2+^, while Ca^2+^ has no inhibiting activity. Increasing concentrations of Zn^2+^ also affected the activity of human HtrA1 and *E. coli* DegP [42], indicating the sensitivity of HtrA proteases towards divalent cations. This shows some disagreement with a previous study, which demonstrates that HpHtrA activity could not be inhibited by high concentrations of bivalent ions (e.g. Mn^2+^, Ca^2+^, or Mg^2+^) [43]. In fact, the activities of HtrA, HhoA (HtrA homologue A), and HhoB (HtrA homologue B) of *Synechocystis* sp. PCC 6803 increased considerably at high calcium ion concentrations. The authors of this study speculated that bivalent cations might interact with the substrate or that their binding may directly activate the HtrA proteases [44]. Therefore, further studies are necessary to reveal possible differences in the regulation of HtrAs from different species.

From our experiments, we conclude that Ca^2+^ has the ability to mask the HtrA signature sites by binding to the calcium-binding motifs within the E-cadherin molecule. The calcium-binding motifs are located between the individual EC domains and play important roles in the adhesive function of E-cadherin [7, 45]. E-cadherin depleted of calcium exhibits a bended structure, which might expose the HtrA signature sites (Fig. 7, open arrows). Binding of calcium to these motifs elongates the curved E-cadherin structure, forming a three-dimensional extracellular structure of adherens junctions through homophilic interactions of the ectodomains in *cis* and *trans* (Fig. 7) [45, 46]. In this complex organized network of E-cadherin molecules, the HtrA cleavage sites are less accessible. This model is supported by our observation that the addition of low calcium ion concentrations efficiently blocked HtrA-mediated E-cadherin cleavage, but did not affect the proteolytic activity of HtrA. Confirming this observation, removal of calcium ions by EDTA considerably increased E-cadherin fragmentation in vitro and on epithelial cells. Although it remains unclear whether inhibition of HtrA-mediated E-cadherin cleavage is a direct consequence of covering the HtrA signature sites for HpHtrA or due to conformational changes within the E-cadherin proteins, these data suggest that calcium binding to E-cadherin diminishes the accessibility of the signature sites to HtrA. This hypothesis is supported by the observation that calcium chelation by EDTA or EGTA led to a defined cleavage pattern of ~90 kDa, ~60 kDa, and ~50 kDa fragments, reflecting the different length of EC5-containing ectodomains of E-cadherin after cleavage of the individual EC tandem repeats starting at the N-terminus (Fig. 7) [35]. Calcium is required in multiple vital cell functions including attachment, morphology, metabolic processes, signal-transduction, replication, etc. [3, 5, 8, 9, 39]. However, whether calcium application relieves *H. pylori* pathogenesis in patients is questionable as *H. pylori* can also activate calcium-dependent E-cadherin-cleaving metalloproteases [27, 41], which can counteract the loss of HtrA-mediated E-cadherin cleavage.

In conclusion, calcium-dependent cell adhesions are crucially important for the physiological function of an intact epithelium [12]. HtrA is an essential protein and constitutively expressed and secreted by *H. pylori* [38]. HpHtrA is certainly important for bacterial physiology, but it has an additional function in infections through its capability of directly cleaving E-cadherin. Since *H. pylori* colonizes the gastric epithelium, we hypothesize that calcium stabilizes E-cadherin junctions and limits HtrA-mediated E-cadherin cleavage during infection.

## Methods

### Cell culture and infection experiments

The gastric epithelial NCI-N87 cells (ATCC, CRL-5822) were grown in RPMI 1640 medium containing 4 mM glutamine (Invitrogen) and 10 % FCS (Sigma) in a humidified atmosphere at 37 °C. The *H. pylori* wild-type strain *Hp*26695 was cultured on agar plates containing 10 % horse serum under microaerophilic conditions for 48 h at 37 °C before infection experiments. Cells were infected with *H. pylori* at a MOI of 100 for 16 h. To investigate the influence of calcium ions or EGTA on *H. pylori*-mediated E-cadherin cleavage, infections were performed in the presence of indicated concentrations of calcium ions or EGTA. Cells were harvested in lysis buffer (20 mM Tris pH 7.5, 1 mM EDTA, 100 mM NaCl, 1 % Triton X-100, 0.5 % DOC, 0.1 % SDS, 0.5 % NP-40). Samples were centrifuged for 10 min at 16,000×*g* at 4 °C to prepare whole cell lysates. Supernatants of infected cells were collected for the detection of the soluble extracellular E-cadherin fragment.

### Recombinant proteins

Recombinant E-cadherin (Asp155-Ile707, Acc. No. NP_004351) was obtained from R&D Systems. According to the manufacturer's instructions, lyophilized rCdh1 was reconstituted in Dulbecco’s phosphate buffered saline (PBS) containing MgCl_2_ and CaCl_2_ (Sigma-Aldrich), resulting in a stock concentration of 100 ng/µl rCdh1, 0.9 mM CaCl_2_, and 0.5 mM MgCl_2_. Production of recombinant HtrA (HpHtrA, Gly18-Lys475, UniProt G2J5T2) wildtype and the inactive HtrA (Ser221 → Ala) from *H. pylori* strain 26,695 has been described elsewhere [36]. Briefly, the GST-tagged HtrA protein was overexpressed in *E. coli* strain BL21. After lysis and binding of the fusion protein to GSH-Sepharose (GE-Healthcare), the GST-tag was removed by incubation with 180 U PreScission™ protease (GE-Healthcare). After purification, the protein was dialyzed against 50 mM Tris (pH 7.5). Purity of HpHtrA was determined by mass-spectrometry [36] and is routinely analyzed by SDS PAGE (92 %). Casein was obtained from Roth (Germany) and reconstituted in H_2_O.

### *In vitro* cleavage assays, SDS-PAGE and western blot

For in vitro cleavage assays, 100 ng rCdh1 was incubated with 200 ng of HpHtrA in 50 mM HEPES, pH 7.5. The reaction volume of 20 µl contained a final concentration 45 µM CaCl_2_ and 25 µM MgCl_2_. If not stated otherwise, the in vitro cleavage reaction was incubated at 37 °C for 16 h. The proteins were separated using SDS-PAGE and blotted onto a nitrocellulose membrane. An antibody recognizing the EC5 domain (Santa Cruz, H108) was used to detect E-cadherin and a polyclonal serum was used to detect HpHtrA as described previously [27]. Alternatively, 10 µg of casein (Roth, Germany) was incubated with 200 ng of HpHtrA at 37 °C for 4 h in combination with indicated concentrations of CaCl_2_, or EGTA. The proteins were separated using SDS-PAGE and visualized using Coomassie G250 (Roth, Germany).

### Non-reducing SDS PAGE and casein zymography

HpHtrA was incubated in 50 mM HEPES (pH 7.5) together with increasing concentrations of CaCl_2_ as indicated. Non-reducing SDS sample buffer without β-mercaptoethanol was added and samples were separated by SDS PAGE containing 0.1 % casein (Roth). Afterwards, the gel was renatured in 2.5 % Triton-X-100 for 2 × 30 min and subsequently equilibrated in developing buffer (50 mM Tris–HCl, pH 7.4, 200 mM NaCl, 5 mM CaCl_2_, 0.02 % Brij35) [36] to refold proteins at 37 °C for 16 h under gentle agitation. Caseinolytic activity in zymograms was visualized by staining with 0.5 % Coomassie Blue R250. To detect oligomers by SDS-PAGE, HtrA was separated as described for zymograms excluding casein as a substrate. All experiments were repeated at least three times.

### Protease activity assay using FITC-labeled casein

Quantification of HpHtrA activity was performed using a fluorescent protease assay kit (Pierce, Thermo Scientific). Trypsin was included as a standard ranging from 0.008 to 1 µg/ml and compared to 1 µg/ml HpHtrA. Where indicated, 0.5 mM CaCl_2_ was added. The measurements were performed in a white, flat bottom 96-well plate (Nunc) at 37 °C. The fluorescence was measured in a plate reader (Infinite^®^ 200 PRO, TECAN) with a filter setting of 485 nm/535 nm (Ex/Em). Statistical analysis was performed using the Student´s *t*-test (paired, two-tailed). Three independent experiments containing three technical replicates were analyzed for every sample. p values >0.05 were not considered statistically significant.

## Additional file

10.1186/s13099-016-0112-6 Supplementary information.

## Abbreviations

Cdh1: E-cadherin
EC: extracellular domain
EDTA: ethylenediaminetetraacetic acid
EGTA: ethyleneglycol-bis-tetraacetic acid
*Helicobacter pylori*: *H. pylori*
HtrA: high temperature requirement A
IC: intracellular domain
TD: transmembrane domain
