# Supplementary material for: Calcium binding protects E-cadherin from cleavage by Helicobacter pylori HtrA
Source: Gut Pathog. 2016 Jun 6;8:29. doi: 10.1186/s13099-016-0112-6 (PMC4895972; doi:10.1186/s13099-016-0112-6)

Supplementary information

Calcium binding protects E-cadherin from cleavage by *Helicobacter pylori* HtrA

Thomas P. Schmidt[1], Camilla Goetz[1], Markus Huemer[1], Gisbert Schneider[2], Silja Wessler[1,#]

Figure legends

Figure S1. Calcium ions, but not magnesium ions change the HtrA-mediated E-cadherin cleavage pattern. (A) Human E-cadherin (hCdh1) was incubated with HpHtrA wild type (+) or with the proteolytic inactive HpHtrA (SA). Where indicated 100 µM $CaCl_2$, 100 µM $MgCl_2$, 125 µM EDTA or 125 µM EGTA was included. Full-length E-cadherin ($rCdh1^{FL}$) and rCdh1 fragments were detected by Western blot using a specific antibody. HpHtrA was probed using a polyclonal antibody to show equal protein loading. (**B**) 10 µg casein were incubated with 1 µg recombinant HpHtrA and, as indicated, with 1 mM $CaCl_2$ or $MgCl_2$ or 16 h at 37°C. Samples were separated by SDS PAGE and proteins were stained by Coomassie G250.

Figure S2: Original images of coomassie-stained SDS gels as shown in Fig. 2. Analysis of casein cleavage by HtrA in the presence of increasing concentration of $CaCl_2$ (**A**) and EDTA (**B**).

Supplement Figure 1

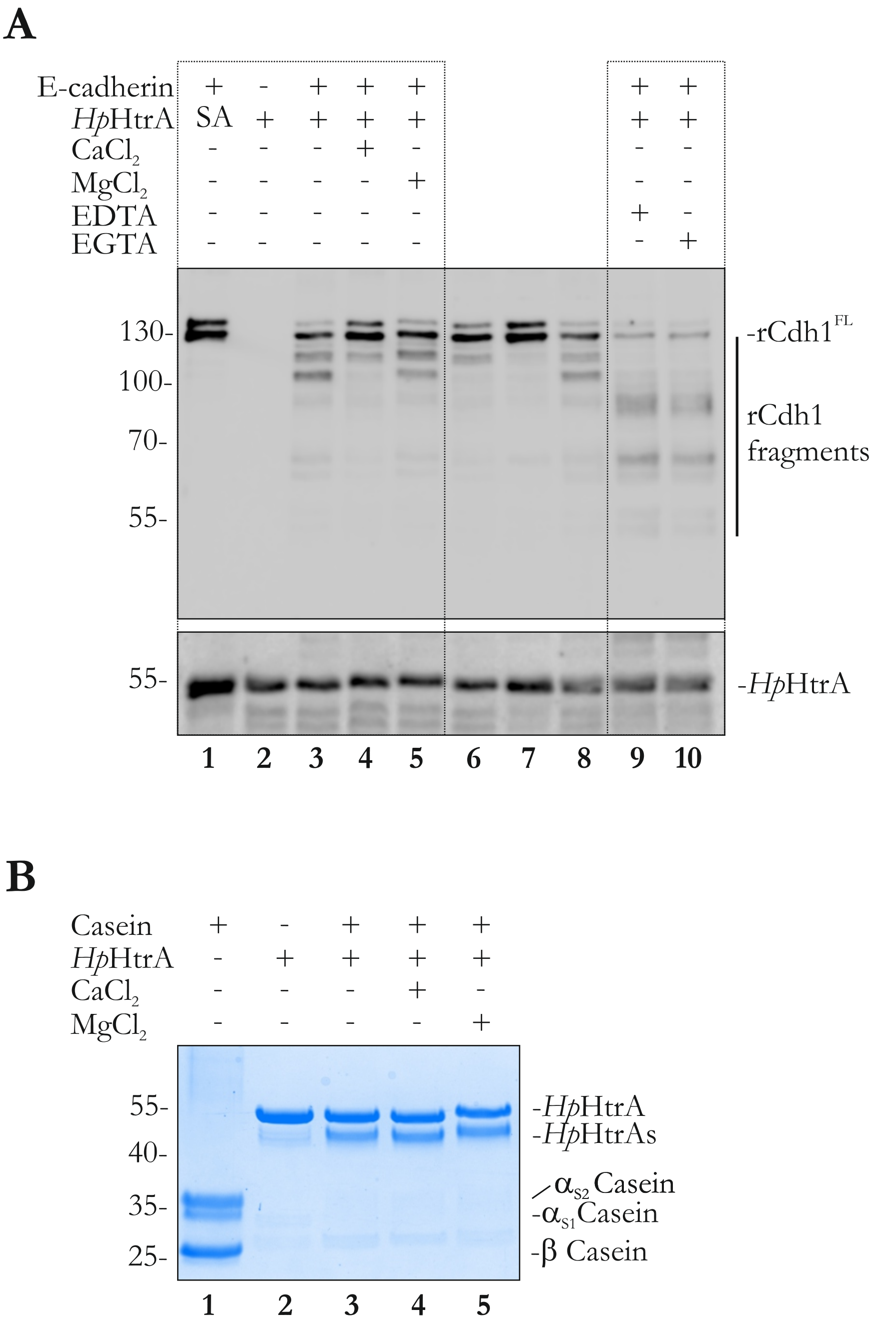

Supplement Figure 2

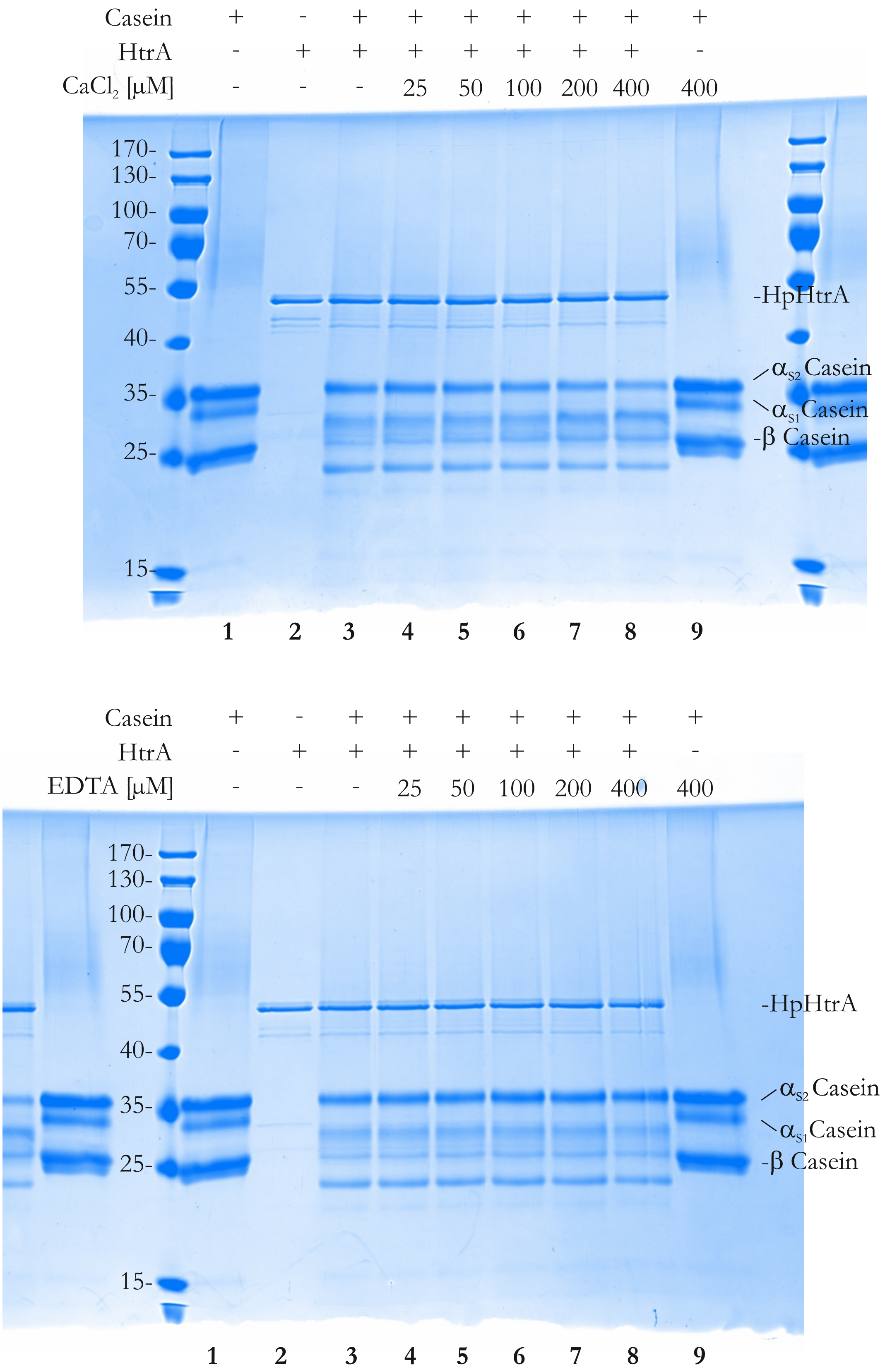

Supplement: Supplementary file 1 — 10.1186/s13099-016-0112-6 Supplementary information. [file 13099_2016_112_MOESM1_ESM.pdf]
